# Sorting through life: evaluating patient-important measures of success in a medication for opioid use disorder (MOUD) treatment program

**DOI:** 10.1186/s13011-022-00510-1

**Published:** 2023-01-14

**Authors:** Megan K. Reed, Kelsey R. Smith, Francesca Ciocco, Richard W. Hass, Avery Lin Cox, Erin L. Kelly, Lara C. Weinstein

**Affiliations:** 1 Department of Emergency Medicine, Sidney Kimmel Medical College, Thomas Jefferson University, 1015 Walnut Street, Curtis Building, Suite 704, PA 19107 Philadelphia, USA; 2 Center for Connected Care, Sidney Kimmel Medical College, Thomas Jefferson University, Philadelphia, PA USA; 3 Jefferson College of Population Health, Thomas Jefferson University, Philadelphia, PA USA; 4 Department of Family and Community Medicine, Sidney Kimmel Medical College, Thomas Jefferson University, Philadelphia, PA USA; 5 Jefferson Center for Interprofessional Practice and Education, Thomas Jefferson University, Philadelphia, PA USA

**Keywords:** Medication for opioid use disorder, Office-based opioid treatment, Pilesorting

## Abstract

**Background:**

Medication for opioid use disorder (MOUD) is the gold standard treatment for opioid use disorder. Traditionally, “success” in MOUD treatment is measured in terms of program retention, adherence to MOUD, and abstinence from opioid and other drug use. While clinically meaningful, these metrics may overlook other aspects of the lives of people with opioid use disorder (OUD) and surprisingly do not reflect the diagnostic criteria for OUD.

**Methods:**

Authors identified items for a pilesorting task to identify participant-driven measures of MOUD treatment success through semi-structured interviews. Interviews were transcribed verbatim and coded in Nvivo using directed and conventional content analysis to identify measures related to treatment success and quality of life goals. Participants of a low-threshold MOUD program were recruited and asked to rank identified measures in order of importance to their own lives. Multidimensional scaling (MDS) compared the similarity of items while non-metric MDS in R specified a two-dimensional solution. Descriptive statistics of participant demographics were generated in SPSS.

**Results:**

Sixteen semi-structured interviews were conducted between June and August 2020 in Philadelphia, PA, USA, and 23 measures were identified for a pilesorting activity. These were combined with 6 traditional measures for a total list of 29 items. Data from 28 people were included in pilesorting analysis. Participants identified a combination of traditional and stakeholder-defined recovery goals as highly important, however, we identified discrepancies between the most frequent and highest ranked items within the importance categories. Measures of success for participants in MOUD programs were complex, multi-dimensional, and varied by the individual. However, some key domains such as emotional well-being, decreased drug use, and attendance to basic functioning may have universal importance. The following clusters of importance were identified: emotional well-being, decreased drug use, and human functioning.

**Conclusions:**

Outcomes from this research have practical applications for those working to provide services in MOUD programs. Programs can use aspects of these domains to both provide patient-centered care and to evaluate success. Specifics from the pilesorting results may also inform approaches to collaborative goal setting during treatment.

## Introduction

Opioid overdose deaths in the US surpassed 80,000 in 2021 [8]. Medication for opioid use disorder (MOUD) with methadone, buprenorphine, or naltrexone is the gold standard evidence-based treatment for opioid use disorder (OUD) [32, 33, 38]. MOUD is associated with longer retention in treatment [44] as well as decreased opioid use [29, 40] and mortality from overdose [48]. Other research suggests MOUD can reduce all-cause mortality and increase quality of life [11, 29]. However, access to and retention in MOUD remains limited in some settings [4, 13, 24]. Improving treatment engagement and outcomes requires consideration of misalignment between the programmatic and patients’ goals of treatment [19, 34].

Traditionally in the literature, “success” in MOUD treatment is measured in terms of program retention, adherence to MOUD, and abstinence from opioid and other drug use (e.g., cocaine, methamphetamine) [5, 20, 27, 31]. While clinically meaningful, these metrics may overlook other aspects of the lives of people with OUD and surprisingly do not reflect the diagnostic criteria for OUD, which include the highly personal repercussions of opioid use such as problems fulfilling obligations, recurring interpersonal problems, and giving up activities [2]. This is important because, as compared to people who are not using opioids, people who use opioids rate their quality of life lower in multiple areas across studies including social functioning, physical and emotional role limitations, general health, and mental health [11, 16]. MOUD treatment may also be considered successful, therefore, if participants report improvements in these areas of quality of life.

Some research on MOUD outcomes increasingly focuses on measuring changes in quality of life among people with OUD. Authors of a recent systematic review reported 12 domains of importance to people with OUD. While these included traditional metrics, such as treatment retention and abstinence from other drugs, they also placed importance on improvements in daily life, physical health, and discontinuing MOUD [42]. Authors called for deeper qualitative investigation into outcomes important to patients.

Hooker et al. [21] reported seven themes of program success for patients that included improvements in physical and mental health, goal-setting, and social relationships [21]. These studies, however, did not ask MOUD participants to rank items within themes, or the themes themselves, in terms of importance. Most studies have been conducted in settings outside of the United States, with different MOUD regulations and program structures [9, 18, 30, 39, 42, 43]. Two studies in the United States focused exclusively on participants in methadone programs [17, 36]. Without knowing outcome importance across multiple themes, MOUD programs will have difficulty establishing priorities with clients. In the present study, we report on ranked outcomes of participant-driven measures of success, gathered through MOUD program participants and the literature, within a multi-site, low-threshold MOUD program.

## Materials and methods

This exploratory study had two arms of data collection: 1) the identification of items for the pilesorting task through semi-structured interviews and 2) the completion of the pilesorting task and collection of relevant participant demographic data. Pilesorting is a mixed-methods approach in which participants are given a stack of terms or domains and asked to sort them into meaningful ranks (e.g., in terms of importance) [46]. This study was designed, conducted, and analyzed by a cisgender female Assistant Professor with an MPH and PhD in Public Health (MKR), a cisgender female research coordinator with a BA in Anthropology (KRS), a cisgender female third-year medical student with a BS in Biology (FS), a cisgender male Assistant Professor with a PhD in Psychology (RH), a cisgender female second-year medical student with a masters in public health (ALC), a cisgender female Assistant Professor with a PhD in Psychology (ELK), and a cisgender female Associate Professor with an MPH, DrPH, and MD (LCW). Authors MKR, KRS, RH, ELK, and LCW all have previous experience with mixed-methods study design, implementation, and analysis. This research was approved by the Institutional Review Board at Thomas Jefferson University. Authors used the consolidated criteria for reporting qualitative research (COREQ) [45].

### Setting and program description

Project HOME Health Services (PHHS) offers low-threshold MOUD services at three locations in Philadelphia, PA, USA 1) Stephen Klein Wellness Center (SKWC), a large primary-care clinic with integrated, on-site behavioral health, dental, and pharmacy services 2) Pathways to Housing PA (PTHPA), an embedded primary care clinic within a Housing First organization for people with experiences of chronic homelessness, serious mental illness, and substance use disorders, and 3) The Hub of Hope, a primary care clinic within a drop-in service center for people experiencing homelessness (for more details see [49]). We extended data collection to include participants of PTHPA MOUD groups, which also included people taking methadone.

### Study design and analysis – qualitative interviews

Items for the pilesorting task were identified through a secondary analysis of qualitative interviews with participants in MOUD programs. The interviews were conducted with potential participants of a community advisory board and demographic data were not collected. As part of the semi-structured interview, patient-important measures of success were captured from participants within a singular MOUD program at the Pathways to Housing PA (PTHPA) site. Staff at PTHPA recruited participants between June to August 2020 and author ELK conducted all interviews. Individuals who were 18 years and older, English-speaking, and an active patient within the MOUD program at Pathways to Housing PA were eligible to participate. Potential participants were given all relevant study information, verbally consented, and asked to complete an interview with ELK over the phone or via Zoom. Virtual data collection was selected because of the COVID-19 pandemic. Participants who completed data collection over Zoom used an iPad in a private conference room in PTHPA. Sixteen interviews were conducted using an interview guide developed by authors LCW and ELK that assessed the following domains of interest: previous experience in MOUD program (“How has it been going in the medication-assisted treatment (MAT)/Suboxone program?”), motivation for treatment (“Why did you decide to start this Suboxone program?”, “Why do you continue to participate in this suboxone program?”), personal goals within MOUD program (“Do you have goals for yourself around Suboxone and drug use?”), and self-identified measures of success (“What does a good day look like for you?”, “How can we tell that people are doing well in this program?”). We used the language in prompts that participants were most familiar with (e.g., “MAT” instead of “MOUD”). Most interviews lasted 20–30 minutes; the shortest was 12 minutes and the longest was 44 minutes. At the completion of the interview, participants were compensated $20 and asked if they would be interested in joining a Patient Advisory Council for the MOUD program. Items from the interviews were supplemented with traditional outcome measures as traditionally used in MOUD program evaluations to create a final list of items for the pilesorting task (see Table 1).

**Table 1:** List of terms and the frequency of bucket assignment from participants in MOUD programs in Philadelphia, PA (*n* = 28). Bold items indicate the highest frequency bucket for each item. Asterisks indicate highest frequency item within the bucket

		Importance Bucket			
Map Number	Item	High	Medium	Low	No
1	Taking my Suboxone as prescribed	**19**	5	4	0
2	Stopping taking Suboxone someday	**10**	8	5	5
3	Abstinence from all drugs	**16**	2	6	4
4	Not using opioids	**23**	2	1	2
5	Decreasing how much I use opioids	**15**	4	2	7
6	Not using other substances that are not opioids (ex. cocaine, methamphetamines, alcohol)	**15**	6	4	3
7	Decreasing how much I use substances that are not opioids (ex. cocaine, methamphetamines, alcohol)	**16**	3	3	6
8	Not being physically dependent on drugs (ex. not needing to get well/not having withdrawal)	**23**	3	1	1
9	Coping with difficult emotions (ex. finding ways to manage when I feel anxious of depressed)	**16**	8	4	0
10	Handling everyday frustrations (ex. not letting little things ruin my day)	**12**	**12**	3	1
11	Being happy	**22**	5	1	0
12	Having hopes, dreams, and goals for the future (ex. feeling hopeful/optimistic about the future)	**21**	2	4	1
13	Having self-worth (ex. feeling good about myself on a daily basis)	**20**	6	2	0
14	(Re)connecting with family	**13**	5	4	6
15	Feeling a part of a community	2	17*	6	3
16	Being able to honestly communicate with others in MAT/Suboxone group	**11**	10	5	2
17	Regularly contributing in MAT/Suboxone group (ex. talking and sharing in group)	**11**	10	5	2
18	Regularly attending MAT/Suboxone group	10	**12**	3	3
19	Accomplishing daily goals/tasks (ex. having a good routine)	**17**	10	1	0
20	Feeling neat and clean (ex. being able to shower/use deodorant/brush my teeth, etc)	**22**	5	1	0
21	Managing my money well (ex. having savings and/or wisely spending my money)	**17**	8	0	3
22	Having a safe, stable place to live	25*	0	2	1
23	Having a safe, stable job	**13**	4	5	6
24	Having a urine test free of drugs	**14**	9	1	4
25	Not getting arrested or violating my probation	**20**	1	2	5
26	Being tested for HIV/HCV infection	8	8	2	10*
27	Decreasing how often I go to the hospital or Emergency Room	7	6	7*	**8**
28	Decreasing how often I overdose	9	3	6	10*
29	Having less physical pain	**12**	9	6	1

Audio-recorded interviews were professionally transcribed verbatim and uploaded into qualitative data analysis software, Nvivo (released in March 2020). Interviews were analyzed by authors MKR, KRS, ELK, and LCW using a combination of directed and conventional content analysis to identify themes related to operationalizing treatment success and quality of life goals [23]. Four coders (authors ELK, MKR, KRS, and LCW) used a combination of a priori codes (e.g., “abstinence from opioids”) and open coding (e.g., “happiness”) to establish a codebook, then coded material in teams of two to three using an iterative process to identify themes related to MOUD treatment success. The team met weekly to discuss progress and resolve discrepancies by consensus. The team reviewed the themes and developed items for pilesorting using participants’ own words when possible (e.g., “Well, when people come, they share. They come every week and they share. You can tell by their sharing how well a person is doing” was grouped with similar comments to make the pilesorting card “Regularly contributing in MAT/Suboxone group”. Saturation for the initial study was achieved as determined by a lack of new domains raised in the three final interviews.

### Study design and analysis - pilesorting

The pilesorting task was facilitated by authors MKR, KRS, and ALC between June and September 2021 at all three PHHS sites. Individuals were recruited by authors at MOUD program meetings and through staff referral at each community site. Individuals who were 18 years and older, English-speaking, and a patient within the MOUD program at their respective community site were eligible to participate. Eligible participants were given all relevant study information, verbally consented, and asked to fill out a demographic survey in a private space prior to beginning the pilesorting task. The demographic survey asked for data on participants’ age, race, ethnicity, gender, education level, housing status, history of substance use and treatment, and engagement with their current MOUD program.

**Fig. 1:**
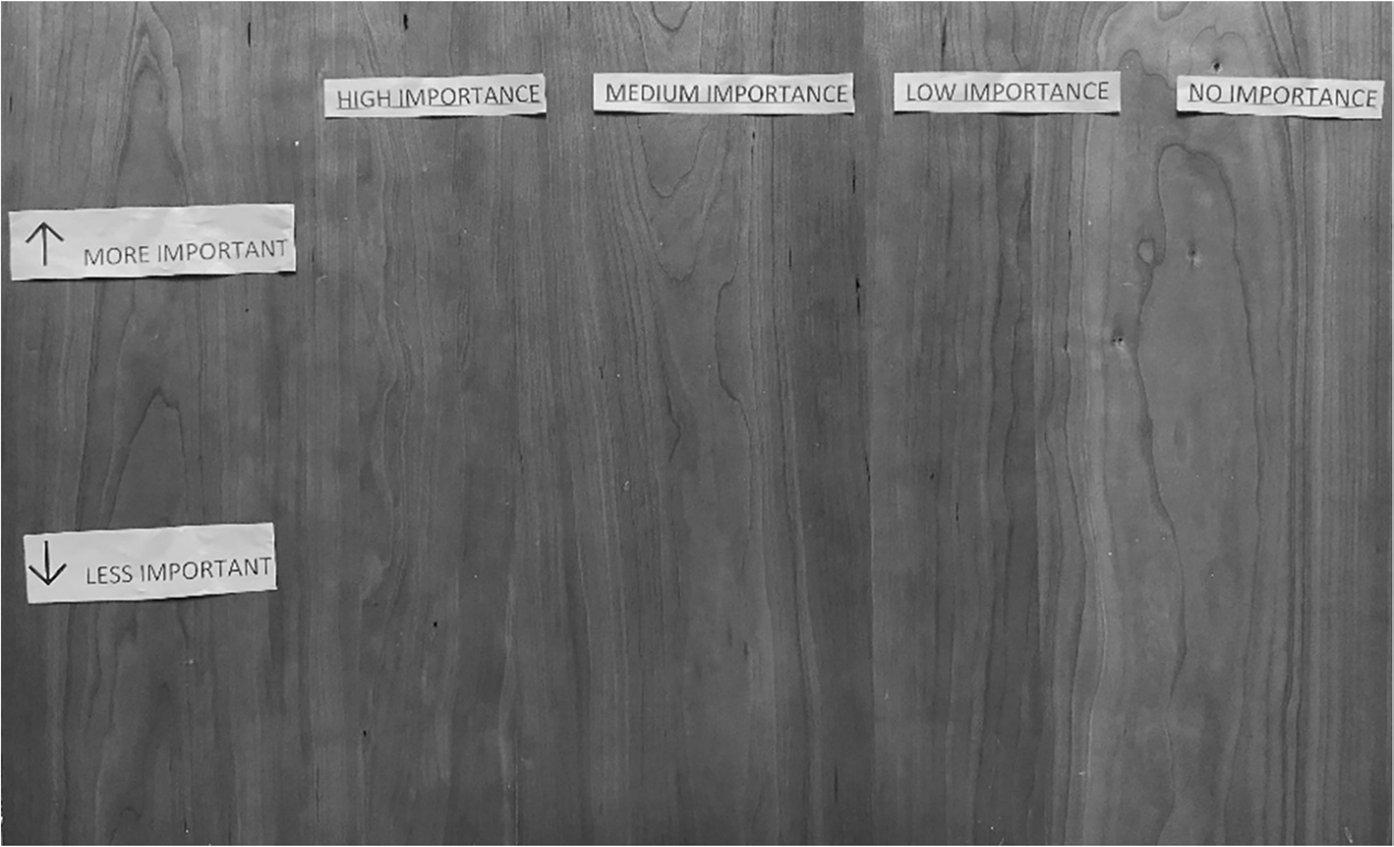
Visual of Pile-Sorting Task Activity. *Note:* Participants were able to place items under each bucket and then to rank order those items within each category of importance

The interviewer then laid out the category titles out on a table in front of individual participants (see Fig. 1) and led with the following prompt: “Imagine it is 5 years from now, we run into each other on the street or in the grocery store. I see you and say ‘[Name of participant], it is good to see you! How are you doing?’ and you respond, ‘I’m doing great, everything is going so well’. We want to know how important each of these things are when you think about doing well or being successful in life or treatment”.

The interviewer then handed the pile of cards to the participant and asked them to sort the cards into the four categories of *High*, *Medium*, *Low*, and *No Importance* without worrying about the order in each individual category. Once the participant had sorted each card into a pile, they were asked to then rank the cards from Most to Least Important within each category. Upon completion of the task, the interviewer thanked the participant and asked if there were any cards that were not in the pile that they thought should be there. These answers were recorded by the interviewer. Participants were compensated with $10 cash and the interviewers recorded the results in Qualtrics using the pick, sort, and rank function. Cards were always returned to the same order for the next participant. The process lasted from 15 to 30 minutes depending on how quickly the participant sorted each card.

Multidimensional scaling (MDS) is the method of choice for pilesorting data analysis [1, 6]. MDS is a data reduction technique in which a matrix of inter-item distances or similarity scores is projected onto a smaller dimensional surface. For example, items might be 15 cities and distances might be miles between each pair of cities. A two-dimensional solution applied to the matrix of inter-city distances will yield coordinates that can be plotted on a two-dimensional map. The map will accurately represent the distances and clustering of the 15 cities.

When items are conceptual, as in the current study, non-metric MDS can be used on a matrix of inter-item similarities. Borghati [6] reasoned that the greater number of participants who put two items in the same pile, the greater the similarity of the items (see also [1]). Thus, the overall inter-item similarity matrix was constructed by first constructing single-subject matrices. The single subject matrices were constructed as follows: a 29 by 29 item matrix with the items in Table 1 as rows and columns was filled with 1’s and 0’s according to whether that participant put the pair of items in the same pile. For example, if a participant put “taking my suboxone as prescribed” and “stop taking my suboxone someday” in the same pile, then the cell in the second row-first column of that single subject matrix would be filled with a 1. These single-subject matrices are symmetric with 1’s on the diagonal, so only the lower triangle is analyzed. Once each participant’s matrix was filled, the 28 single-subject matrices were added together to yield an overall interitem matrix. This overall inter-item similarity matrix represented similarity in terms of the number of participants who put the pair of terms in the same pile. For example, if 28 out of 28 participants put “taking my suboxone as prescribed” and “stop taking my suboxone someday” in the same pile, the overall inter-item matrix would have a 28 in the second row-first column. The process of creating the matrices was automated using the R Statistical Programming Environment [41].

Non-metric MDS was then performed in R on the overall similarity matrix specifying a two-dimensional solution. Prior to running MDS, the matrix was transformed into a dissimilarity matrix by subtracting each cell count from the highest off-diagonal cell count (23 in this case). This yielded a more stable solution and simply re-scaled the item distances while preserving the relationships. The goal of this non-metric MDS was to represent the distances among the items in a two-dimensional space. This kind of dimension reduction aids in interpreting the distances (and similarities) among the items and can yield insights into participants’ conceptualizations of concepts that can be used to make new questionnaires and surveys.

## Results

### Semi-structured interviews

A total of 16 semi-structured interviews were conducted with Pathways to Housing PA MOUD program participants between June and August 2020. No demographic data were collected and therefore, none are reported here. Analysis of themes resulted in items 1–23 in Table 1. An additional six items typically found in MOUD program evaluations (negative urine drug screens, incarceration and recidivism, HIV/HCV infection, hospitalization and Emergency Department utilization, and the reduction of overdose and pain) were added for a grand total of 29 items [3, 7, 11, 12, 14, 15, 25, 26, 28, 29, 40, 44, 49].

### Pilesorting task

Thirty-one people provided informed consent. Among these, two were unable to complete the task and a third was ineligible, leaving a final dataset from 28 people. The median age of participants was 42 years old. Most participants identified as not Latino or Hispanic (96%), White (54%) or Black/African American (43%), and male (71%). Most had a Grade 12 or GED education (54%) and were living in their own home/apartment (57%). Only one participant was on methadone; the remainder (96%) were on buprenorphine The median age of first opioid use was 24. Seventy-one percent considered heroin/fentanyl to be their drug of choice and 46% had used heroin/fentanyl in last 30 days. Fifty-four percent reported taking buprenorphine 7 days a week. (see Table 2) One participant had also participated in the semi-structured interview.

**Table 2:** Demographics and drug use histories of pilesorting study participants (*N* = 28)

Variable	*N* (%)
**Age** (median, interquartile range [IQR])	42 (34, 53)
**Ethnicity**	
Latino or Hispanic	1 (3.6)
Not Latino or Hispanic	27 (96.4)
**Race**	
White	15 (53.6)
Black/African American	12 (42.9)
Other^a^	1 (3.6)
**Gender**	
Male	20 (71.4)
Female	7 (25.0)
Transgender Female	1 (3.6)
**Education**	
Less than High School graduate	4 (14.3)
Grade 12 or GED	15 (53.6)
1–3 years of college	9 (32.1)
College graduate	0 (0)
**Main living situation in the past 30 days**	
My own home or apartment	16 (57.1)
Halfway/three-quarter house or other transitional house	3 (10.7)
A friend’s home	3 (10.7)
A shelter	3 (10.7)
On the street	2 (7.1)
Other^b^	2 (7.1)
Age at first opioid use (median, IQR)^c^	24 (16, 32)
Drug(s) of choice^d^	
Heroin/fentanyl	20 (71.4)
Cannabis	9 (32.1)
Powder cocaine	8 (28.6)
Alcohol	7 (25.0)
Crack cocaine	6 (21.4)
Prescription opioids	3 (10.7)
Benzodiazepines	2 (7.1)
Methamphetamine/amphetamine	1 (3.6)
PCP/Wet/Angel Dust	1 (3.6)
Synthetic cannabinoids (“K2”)	1 (3.6)
Buprenorphine/methadone	1 (3.6)
Ecstasy	1 (3.6)
Past 30-day use^d^	
Nicotine	20 (71.4)
Heroin/fentanyl	13 (46.4)
Cannabis	13 (46.4)
Alcohol	9 (32.1)
Crack cocaine	9 (32.1)
Powder cocaine	7 (25.0)
Benzodiazepines	4 (14.3)
Methamphetamine/amphetamine	1 (3.6)
Prescription opioids	1 (3.6)
PCP/Wet/Angel Dust	1 (3.6)
Synthetic cannabinoids (“K2”)	1 (3.6)
Ecstasy	3 (10.7)
Kratom	1 (3.6)
Program	
Stephen Klein Wellness Center	8 (28.6)
Pathways to Housing PA	13 (46.4)
Hub of Hope	7 (25.0)
Number of days participant took MOUD in the past 7 days	
1	1 (3.6)
2	0 (0)
3	2 (7.1)
4	5 (17.9)
5	4 (14.3)
6	1 (3.6)
7	15 (53.6)
Previous substance use disorder treatment modalities^d^	
Inpatient	23 (82.1)
Outpatient	26 (92.9)
Methadone	16 (57.1)
Naltrexone (Vivitrol)	4 (14.3)
Another buprenorphine program	12 (42.9)
AA/NA/other abstinence-based group	19 (67.9)
Individual therapy	19 (67.9)
Length of participation in this program	
< 3 months	3 (10.7)
3–6 months	6 (21.4)
6 months - 1 year	4 (14.3)
1–2 years	7 (25.0)
2-3 years	3 (10.7)
> 3 years	5 (17.9)
Communication with Suboxone provider during COVID-19	
More than once a week	3 (10.7)
Once a week	9 (32.1)
3 times a month	5 (17.9)
2 times a month	5 (17.9)
Once a month or less	6 (21.4)

^a^ “Other” includes multiracial Black and White

^b^ “Other” includes “family and shelter”, “renting a room”

^c^ Data missing for 1 participant

^d^ Participants could choose multiple answers, will not sum to 100%

Most reported participation in prior substance treatment programs, including outpatient (93%), inpatient (82%), AA/NA or another abstinence-based group (68%), individual therapy (68%), and methadone (57%). Forty-six percent of participants had participated in the buprenorphine program for 1 year or less. Before COVID-19, most participants were attending a buprenorphine group in person either once a week (32%) or not at all (61%). Since COVID-19, 43% of participants were communicating with their buprenorphine provider once a week or more.

**Fig. 2:**
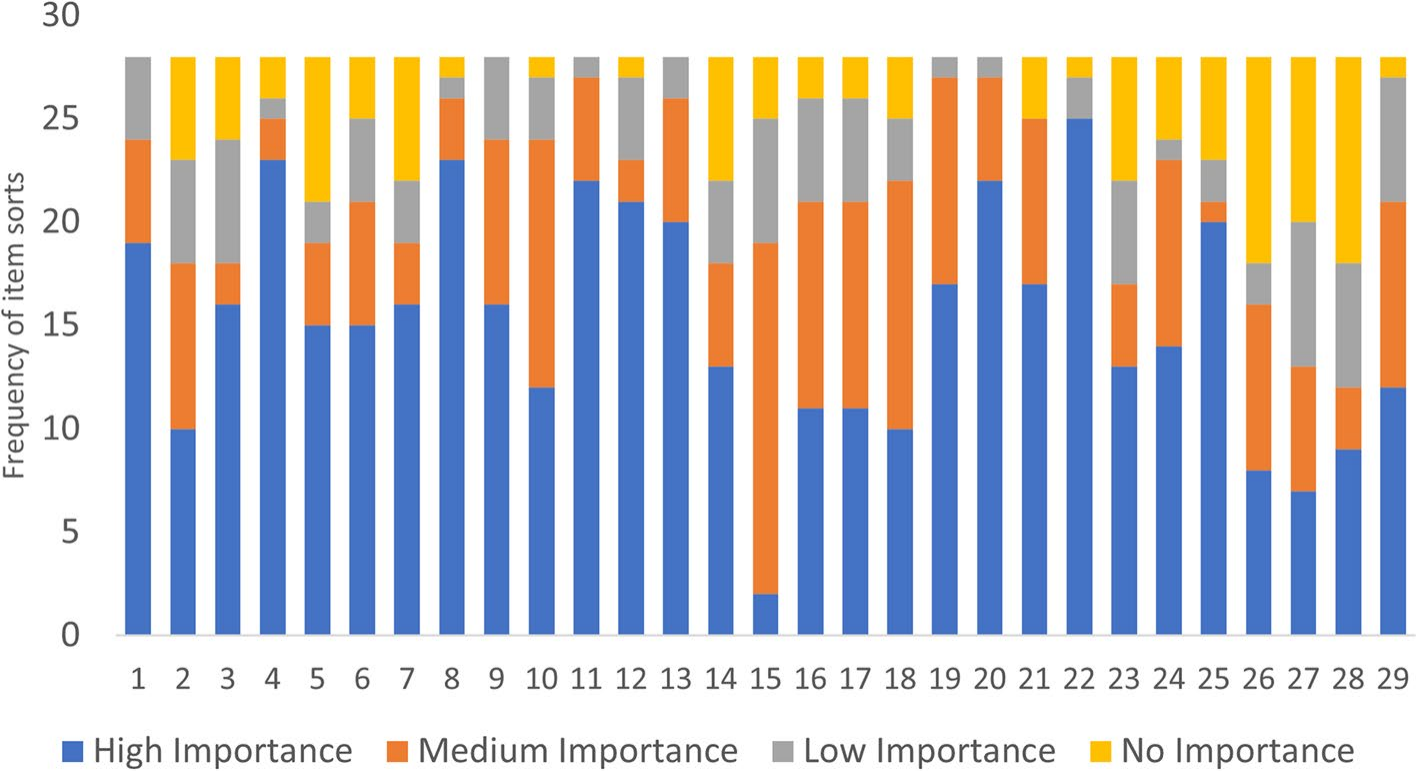
List of term numbers* and the frequency of bucket assignment from participants in MOUD programs in Philadelphia, PA (*n* = 28) *Please see Table 2 to connect term number to corresponding term

Item frequency and rank did not perfectly overlap. Participants most frequently placed the following items in the *High Importance* bucket: stable housing (*n* = 25), no opioids (*n* = 23), no physical dependence (*n* = 23), being happy (*n* = 22), feeling neat and clean (*n* = 22), optimism (*n* = 21), self-worth (*n* = 20), and no arrests (*n* = 20). The most frequently placed items in the *Medium Importance* bucket were having a sense of community (*n* = 17), attending group (*n* = 12), and handling frustrations (*n* = 12). In the *Low Importance* bucket, participants most frequently selected decreasing ED or hospital visits (*n* = 3), decreasing OD (*n* = 6), having a sense of community (*n* = 6), abstinence from all drugs (*n* = 6), and less pain (*n* = 4). In the *No Importance* bucket, the most frequently placed items were being tested for HIV/HCV (*N* = 10), decreasing OD, (*n* = 10) decreasing ED or hospital visits (*n* = 8), and decreasing use of opioids (*n* = 7) (see Fig. 2). By contrast, the highest median ranked items in this *High Importance* bucket were abstinence from all drugs (median rank = 2), stable housing (median rank = 4) not using opioids (median rank = 5) and being happy (median rank = 5). The highest median ranked items in the *Medium Importance* bucket were decreasing opioid use (median rank = 1.5) and (re)connecting with family (median rank = 2). The highest median ranked items in the *Low Importance* bucket were not using opioids (median rank = 1), decreasing opioid use (median rank = 1.5), not using other non-opioid drugs (median rank = 1.5), and (re)connecting with family (median rank = 1.5). The highest median ranked items in the *No Importance* bucket were abstinence from all drugs (median rank = 1) and not being physically dependent on drugs (median rank = 1).

The MDS solution and map are easier to interpret in relation to the ranking of items within each of the importance “buckets.” The importance of items was expressed in terms of *frequency* (e.g., how many participants placed the term in a bucket) and *rank* (e.g., the median location within a bucket where items were placed). Overall, every item (*n* = 29) was placed in the high importance bucket at by at least 1 participant, “Having a stable place to live” the only item to not appear in the medium importance bucket, “Managing money well” was the only item to not appear in the low importance bucket, and while 6 items did not appear in the no importance bucket: “Taking my suboxone as prescribed”, “Coping with difficult emotions”, “being happy”, “having self-worth”, “accomplishing daily goals/tasks”, and “feeling neat and clean.”

**Fig. 3:**
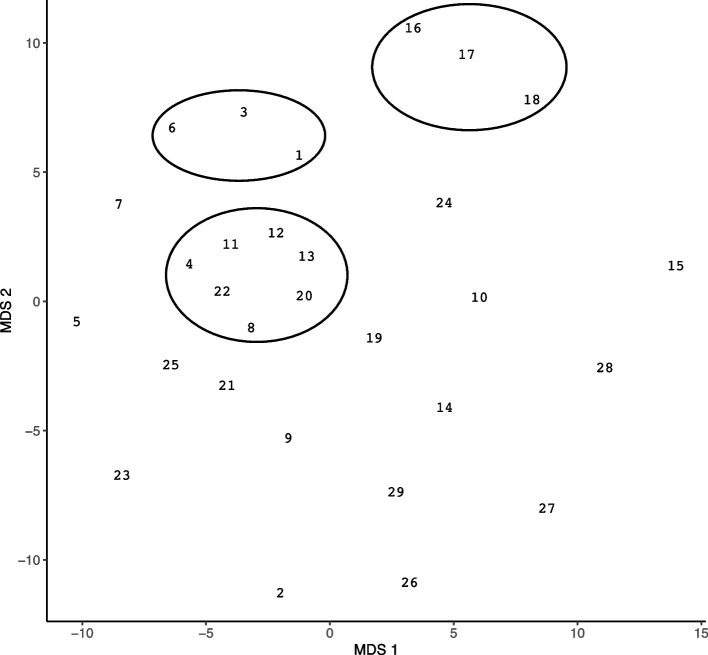
Multi-Dimensional Scaling Map of MOUD Treatment Outcomes. Cluster 1 (Items 16, 17, 18): *Being able to honestly communicate with others in MAT/Suboxone group, regularly contributing in MAT/Suboxone group (ex. Talking and sharing in group), regularly attending MAT/Suboxone group*. Cluster 2 (Items 6, 3, 1): *Not using other substances that are not opioids (ex. Cocaine, methamphetamine, alcohol), abstinence from all drugs, taking my Suboxone as prescribed*. Cluster 3 (Items 4, 11, 12, 13, 22, 8, 20): *not using opioids; being happy; having hopes, dreams and goals for the future (ex. Feeling hopeful/optimistic about the future); having self-worth (ex. Feeling good about myself on a daily basis); having a safe, stable place to live, not being physically dependent on drugs (ex. Not needing to get well/not having withdrawal); feeling neat and clean (ex. Being able to shower/use deodorant/brush my teeth,* etc.*)*. *Note:* The numbers reflect each of the items (see Table 1) and their relative proximity reflects their conceptual clustering, meaning that items that are further apart are less conceptually close

As described, non-metric MDS was used to map the inter-item similarities onto a two-dimensional map (see Fig. 3). Along with frequency information, this allows for an understanding of whether participants see different items as similar in terms of importance. This was done by interpreting closely plotted items as conceptual clusters [22]. Figure 3 illustrates three conceptual clusters of items. These clusters were identified through research team consensus. First, a primary cluster in the middle was comprised of three facets of *emotional well-being* (e.g., optimism, being happy, having a sense of self-worth), *decreased drug use* (e.g., no opioids, no physical dependence), and *human functioning* (e.g., stable housing, being neat and clean). All items within these clusters most often appeared in the high-importance bucket, with stable housing being the item with the greatest number of participants (*n* = 25) sorting it into the high-importance bucket.

Items in the other clusters were more homogenous. In the second cluster were items related to *buprenorphine groups* (e.g., attending group, contributing to the group, and being honest in group). Items in this cluster were more uniformly distributed among the buckets by participants who mostly sorted them into the high and medium frequency buckets. The third cluster was related to *traditional buprenorphine success metrics* (e.g., taking my Suboxone as prescribed, abstinence, no other substances). Items in this cluster were most often sorted into the high importance bucket.

Participants added 18 additional items when asked whether anything important to them and their success was not on the list (*“Are there any items that aren’t here that you think should be?”).* These were categorized through discussion with the entire author group into improved relationships (e.g., having friends, being able to actively support family), personal well-being (e.g., talk therapy, taking care of medical needs, self-accountability), and daily life (e.g., having hobbies not related to drug use, exercise, spirituality).

## Discussion

In this exploratory study, participants in MOUD services ranked the overall priority of participant-derived measures and traditional metrics in order of importance to their own lives and indicators of success from their MOUD treatment. Participants identified a combination of traditional and stakeholder-defined recovery goals as highly important, however, we identified discrepancies between the most frequent and highest ranked items within the importance categories. The main items identified as highly important and ranked highest by participants, which were clustered in the multi-dimensional scaling map, included *stable housing, not using opioids, being happy,* and *having a sense of self-worth.* This indicates that while the traditional goal of not using opioids specifically is of primary importance to PWUD, other facets of life that represent a more holistic sense of recovery are also important. For PWUD, other traditional outcomes of *not being physically dependent on a drug* and *not being arrested* were frequently named as highly important but did not rise to a level of high rank, indicating these might not be relevant current concerns for participants and reflect a point of divergence from providers’ primary priorities for them. These findings are part of a growing literature supporting an expanded understanding of how services providers could evaluate the progress of their participants that is more congruent with the treatment goals of their participants [21, 42].

Some traditional measures of MOUD success, such as being tested for HIV, decreasing overdose, and decreasing hospital visits are often used as measures for “quality of life” for people who use drugs [10, 35, 37, 47]. Our results were not aligned with these measures, nor were they in other recent literature with patient-derived measures of MOUD success [21, 42]. Facets of quality of life such as emotional regulation and housing stability took precedence over health-related variables. It should be noted that housing stability may have been especially important to participants in our study due to the participation of many participants in housing programs. Similarly, participants were recruited from organizations that provided other services to participants (e.g., health care, benefits coordination). If participants were having health-related needs met, they may have ranked related items as lower in importance.

In comparison to US-based studies with methadone program participants, our results had mixed support of findings from Gelpi-Acosta [17] and Mitchell et al. [36]. Participants from the former expressed a wish to avoid withdrawal, cease opioid use, and to have employment and family connections [17]. The latter echoed our findings more closely with participant emphases on stable housing, employment, and abstinence [36].

Unlike findings from some earlier work [36, 43], participants in our sample did not regularly emphasize the importance of a social network and social inclusion. However, responses to the open-ended question about what else comprised success resulted in some relationship-focused items, which could mean that the existing prompts related to social connection were less meaningful to participants. For example, our items indicating social connection were largely about desiring greater connections with others with lived experiences of drug use as opposed to greater connections with non-drug using peers. The open-ended responses, in contrast, included people not connected primarily through sharing a diagnosis of opioid use disorder. Expanding conceptualizations of what are meaningful elements of quality of life is important, as focusing solely on health-related quality of life measures for people with opioid use disorder is reductive, focusing almost exclusively on their lives as it relates to their drug use.

Outcomes from this research may have practical applications for those working to provide services in MOUD programs. In aggregate, participants placed a high number of terms in the *High Importance* category. This reflects that MOUD program participants have multiple, urgent, and likely competing demands. Therefore, choosing treatment priorities may feel difficult and overwhelming. Clinicians and others working with MOUD program participants in unstable circumstances should recognize how these competing demands may interfere with participants’ ability to consistently work towards a subset of goals at a time. Having conversations about how to prioritize these competing demands might help to mitigate these risks in conjunction with low-threshold programming (e.g., not adopting punitive policies for positive urine drug screens or missed appointments).

Specifics from the pilesorting results may inform approaches to collaborative goal setting during treatment. For example, most items about MOUD group were placed in close proximity to one another. To reduce the number of terms presented to clients during intake or goalsetting, a factor analysis can identify latent variables assessing the same domain. Our visual assessment of clusters identified the following latent variables as candidates for future measurement of treatment goals: emotional well-being (e.g., feeling happy and navigating difficult emotions), decreased drug use (e.g., abstinence of reduction of use), and attendance to basic functioning (e.g., being able to take care of daily needs and activities). Presenting these reduced categories to MOUD participants to identify their priority areas represents a truly patient-centered approach to working to achieve a range of life goals. As recommended elsewhere, these can be developed into scales and surveys for use with future patients to inform treatment structure [21, 42].

## Limitations

The terms presented to participants were summarized from descriptions in the qualitative interviews and the literature and may have been interpreted differently by participants. Items from the qualitative interviews were generated by people experiencing chronic homelessness from whom we did not capture demographic data. They may have had different definitions of treatment success than participants at the other sites for the pilesorting activities. For example, unlike earlier work, participants in our sample did not regularly emphasize the importance of a social network and social inclusion [21, 42]. However, our social domains were largely about connections with others with lived experiences of drug use. Supporting the possible need to revise some items, responses to the open-ended question about what else comprised success resulted in some relationship-focused responses with those who are not involved in opioid use, which could mean that the existing prompts related to social connection were less meaningful to participants. Second, the definition of *No Importance* may differ across participants as they could place items in this bucket that were both not meaningful to them personally as well as items that were not applicable to them. For example, someone no longer at risk for HIV would place that item for the same bucket as they would for “contributing to MAT group”, which may not be an important facet if they don’t attend groups. Further, we collected data from a small sample participating in three MOUD programs in Philadelphia, PA, limiting generalizability of results. All but one of our participants was prescribed buprenorphine as MOUD, limiting our ability to generalize results to people on methadone. Our ability to generalize based on sample characteristics (e.g., only eight women) is restricted. A larger sample size could be stratified by participant time in treatment to determine whether those with greater time on MOUD had meaningfully different rankings than those newly enrolled in MOUD. Finally, data were collected during the COVID-19 pandemic, which may have influenced participant responses.

## Conclusion

Measures of success for participants in MOUD programs are complex, multi-dimensional, and vary by the individual. However, some key domains such as emotional well-being, decreased drug use, and attendance to basic functioning may have universal importance. Programs can use aspects of these domains to both provide patient-centered care and to evaluate success.

## Data Availability

Due to the nature of this research, participants of this study did not agree for their data to be shared publicly, so supporting data is not available.
